# Pulmonary alveolar proteinosis and *Pneumocystis jirovecii* infection in an infant with hyper-IgM syndrome caused by a novel *CD40LG* variant: a case report

**DOI:** 10.3389/fped.2026.1913335

**Published:** 2026-08-11

**Authors:** C. Leclercq, E. Olinger, A. Caminoa, O. Chatzis, D. Dumitriu, A. Froidure, J. Smet, S. Balbeur, T. Corbisier

**Affiliations:** 1 Department of Pediatrics, Cliniques Universitaires Saint-Luc, UCLouvain, Brussels, Belgium; 2 Department of Human Genetics, Cliniques Universitaires Saint-Luc, UCLouvain, Brussels, Belgium; 3 Department of Pathological Anatomy, Cliniques Universitaires Saint-Luc, UCLouvain, Brussels, Belgium; 4 Department of Pediatric Radiology, Cliniques Universitaires Saint-Luc, UCLouvain, Brussels, Belgium; 5 Pulmonology Department, Cliniques Universitaires Saint-Luc, UCLouvain, Brussels, Belgium; 6 Department of Immunology, Laboratoire Hospitalier Universitaire de Bruxelles – Universitair Laboratorium Brussel (LHUB-ULB), Université Libre de Bruxelles (ULB), Brussels, Belgium; 7 Immunology Department, University Hospital Brugmann, Université Libre de Bruxelles (ULB), Brussels, Belgium; 8 Department of Pediatric Hematology-Oncology, Institut Roi Albert II, Cliniques Universitaires Saint-Luc, UCLouvain, Brussels, Belgium; 9 Department of Pediatrics, Clinique Saint-Pierre, Ottignies, Belgium; 10 Pediatric Intensive Care Unit, Cliniques Universitaires Saint-Luc, UCLouvain, Brussels, Belgium

**Keywords:** CD40 ligand deficiency, infant, opportunistic infections, *Pneumocystis jirovecii*, pulmonary alveolar proteinosis, X-linked hyper-IgM syndrome

## Abstract

**Background:**

Pulmonary alveolar proteinosis (PAP) is a rare interstitial lung disease. While autoimmunity is the leading cause of PAP in adults, the majority of pediatric cases occur secondary to immunodeficiency and opportunistic infections. Secondary PAP associated with CD40 ligand (CD40L) deficiency is extremely rare, and the underlying pathophysiology remains poorly understood.

**Case presentation:**

We report the case of a 4-month-old boy admitted due to failure to thrive and severe hypoxemic respiratory failure. Chest computed tomography demonstrated diffuse ground-glass opacities with interlobular septal thickening. Bronchoalveolar lavage yielded clear fluid containing Periodic acid-Schiff-positive material consistent with PAP, and polymerase chain reaction was positive for *Pneumocystis jirovecii*. An immunologic evaluation showed hypogammaglobulinemia (IgG and IgA), a normal IgM level for his age, an absence of class-switched memory B cells, and significantly reduced CD40L expression on activated T lymphocytes. Targeted genetic testing identified a previously unreported hemizygous *CD40LG* missense variant [c.137T>C, p.(Leu46Pro)]. Integration of the patient’s immunophenotype, an *in silico* variant evaluation, and segregation data permitted classification of this variant as likely pathogenic, confirming X-linked hyper-IgM syndrome. The patient improved with ventilatory support, high-dose trimethoprim-sulfamethoxazole, and immunoglobulin replacement therapy.

**Conclusions:**

Early-onset PAP associated with *P. jirovecii* infection may reveal CD40L deficiency and should prompt evaluation for primary immunodeficiency. An integrated clinico-immunologic assessment coupled with genetic testing is essential to provide a molecular diagnosis, guide management, and prognostication, and to support timely consideration of curative hematopoietic stem cell transplantation.

## 1 Introduction

X-linked hyper-IgM (XHIGM) syndrome is a rare primary immunodeficiency caused by defective immunoglobulin class-switch recombination. Its estimated prevalence is approximately 1 in 1,000,000 men, and it accounts for 65%–70% of cases of hyper-IgM syndrome (1–3). The syndrome results from hemizygous pathogenic variants in *CD40LG*, which is located on the X chromosome and encodes the CD40 ligand (CD40L), expressed primarily on activated CD4^⁺^ T cells. CD40L is a 32-kDa protein consisting of 261 amino acids, containing a short intracytoplasmic tail, a single transmembrane (TM) α-helix, and an extracellular portion (EC) with a TNF-homology domain (4). Interaction between CD40L and CD40 on B cells and macrophages is essential for B-cell proliferation, class-switch recombination, and somatic hypermutation. Consequently, CD40L deficiency leads to a combined humoral and cellular immunodeficiency. Affected patients typically present with low IgG, IgA, and IgE levels, normal or elevated IgM levels, and preserved total B-cell counts despite a marked reduction in class-switched memory B cells. These patients are predisposed to severe, recurrent, and opportunistic infections, along with autoimmune and malignant complications (3, 5).

Pulmonary alveolar proteinosis (PAP) is a rare form of childhood interstitial lung disease (chILD) characterized by the intra-alveolar accumulation of surfactant, which impairs gas exchange. According to the American Thoracic Society classification, chILD is defined by respiratory symptoms, signs of respiratory insufficiency, hypoxemia, and diffuse parenchymal abnormalities on imaging (6–8). Surfactant accumulation results either from increased production by alveolar type II pneumocytes or from impaired clearance by dysfunctional alveolar macrophages. PAP represents a heterogeneous clinicopathologic entity with diverse etiologies, ranging from primary genetic disorders to secondary forms associated with infections, primary and secondary immunodeficiencies, and autoimmune diseases. While autoimmunity driven by antibodies against granulocyte–macrophage colony-stimulating factor (GM-CSF) is the leading cause of PAP in adults, the majority of pediatric cases are secondary to immune deficiency. Therefore, these causes should be systematically investigated in affected children (9–11).

We report the case of a 4-month-old male infant with PAP associated with invasive *Pneumocystis jirovecii* pneumonia, which revealed an underlying X-linked hyper-IgM syndrome. PAP in the context of CD40L deficiency has only rarely been described, and its pathophysiology remains poorly understood, making this case a relevant contribution to the literature (12–14). We identified a previously unreported *CD40LG* missense variant located in the transmembrane domain of CD40L, classified as likely pathogenic based on the patient's immunophenotype, clinical presentation, variant *in silico* characteristics, and segregation data.

## 2 Case presentation

**Figure 1:**
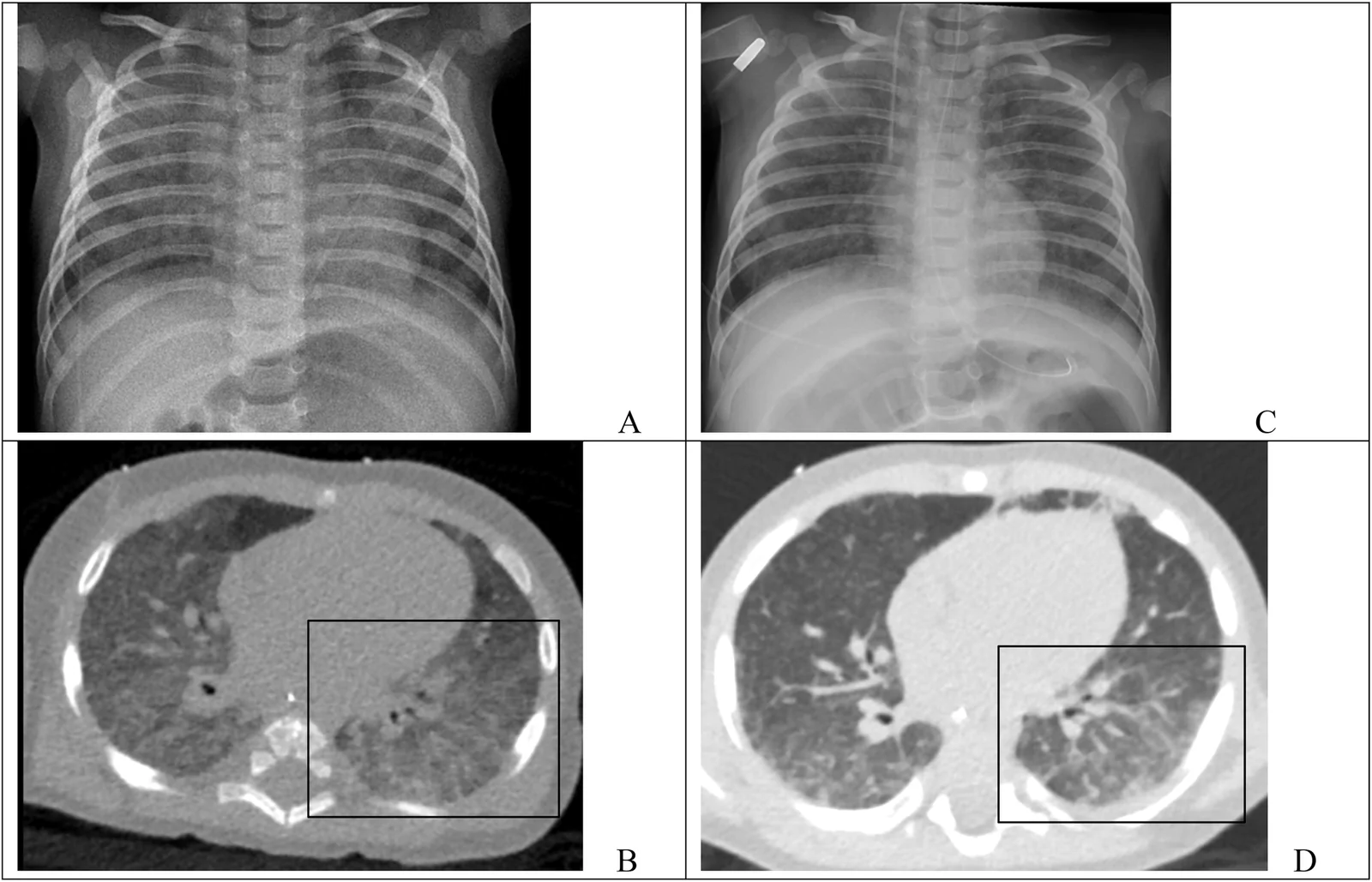
Chest imaging findings. **(A)** Initial chest radiograph (week 1): diffuse ground-glass opacities. **(B)** Initial chest CT (week 1): diffuse ground-glass opacities with interlobular septal thickening, predominantly in the basal regions. **(C)** Follow-up chest radiograph (week 6; 2 weeks post-treatment): near-complete normalization of the pulmonary parenchymal abnormalities. **(D)** Follow-up chest CT (week 6; 2 weeks post-treatment): marked improvement with residual basal ground-glass opacities, predominating in the dependent lung regions, and interstitial thickening.

**Figure 2:**
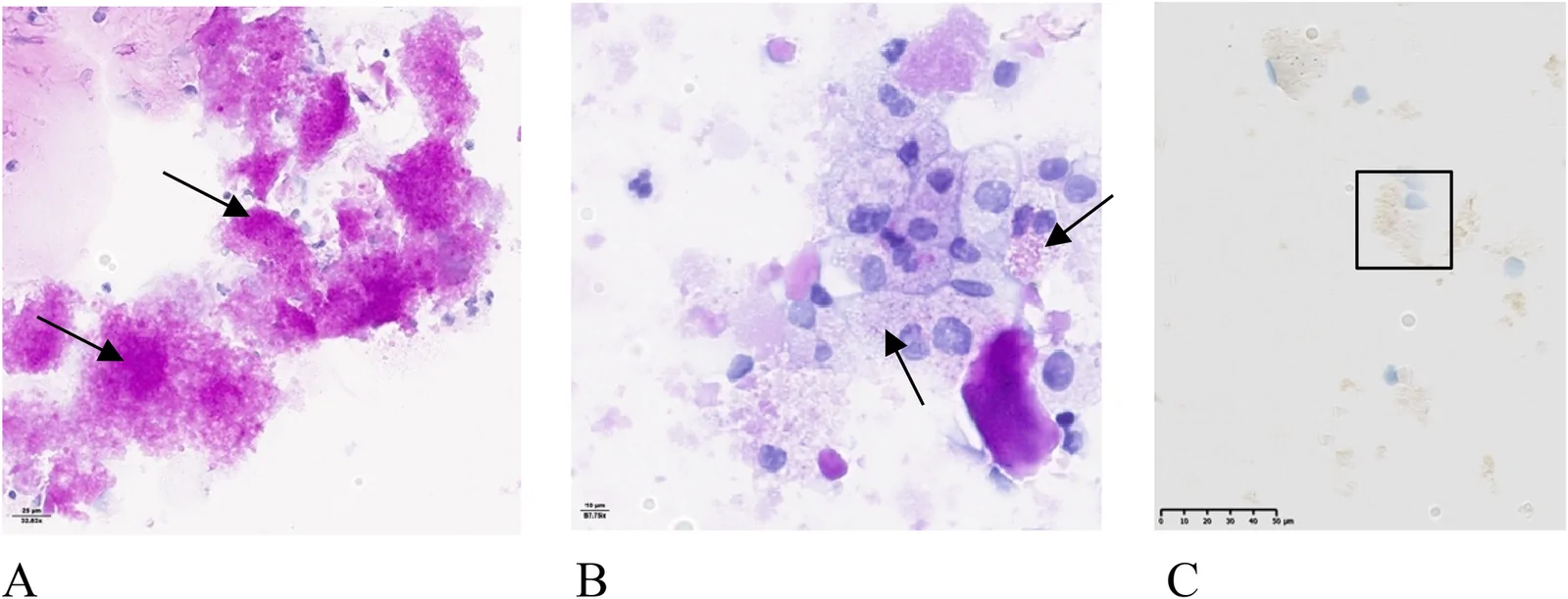
Bronchoalveolar lavage cytology findings. **(A)** *P. jirovecii* infection: fine, foamy, cotton-like, or honeycomb-like alveolar material with identifiable microcysts. **(B)** Pulmonary alveolar proteinosis: denser amorphous proteinaceous material with diffuse PAS positivity and no identifiable organisms. **(C)** *Pneumocystis* immunohistochemistry: weak positivity for microorganisms.

A 4-month-old male infant was admitted to the pediatric intensive care unit due to unexplained hypoxemia associated with failure to thrive and feeding difficulties. Over the preceding month, he had developed afebrile upper respiratory symptoms followed by episodes of cyanosis and sweating occurring primarily during feeding, without syncope. The infant had no relevant past medical history, and he was born at term with normal neonatal adaptation. His parents were non-consanguineous and of white ethnicity. Family history was unremarkable. Upon admission, the patient presented with tachypnea and severe hypoxemia (oxygen saturation of 70%) requiring low-flow oxygen therapy. Cardiac and pulmonary auscultation were unremarkable. A growth assessment showed a stagnation in weight gain over the previous month. Chest radiography demonstrated diffuse ground-glass pulmonary opacities (Figure 1A). Laboratory evaluation showed normal C-reactive protein levels, a normal leukocyte count, and a normal total lymphocyte count for his age. Transthoracic echocardiography showed normal cardiac morphology and function. Chest computed tomography (CT) revealed diffuse and homogeneous ground-glass opacities associated with interlobular septal thickening, predominantly in the basal regions, without pulmonary volume loss or abnormalities of the bronchovascular structures (Figure 1B). Bronchoalveolar lavage (BAL) fluid was clear. A cytological examination showed a background of cellular debris with numerous ballooned macrophages, lymphocytes, neutrophils, eosinophils, and bronchial epithelial cells. In addition, extracellular flocculent aggregates composed of spherules were identified, raising suspicion for *Pneumocystis* infection, which was confirmed by positive immunostaining. Periodic acid-Schiff (PAS) staining was also positive, showing dense amorphous proteinaceous material consistent with PAP (Figure 2). Given these findings, an etiological investigation for PAP was initiated.

**Figure 3:**
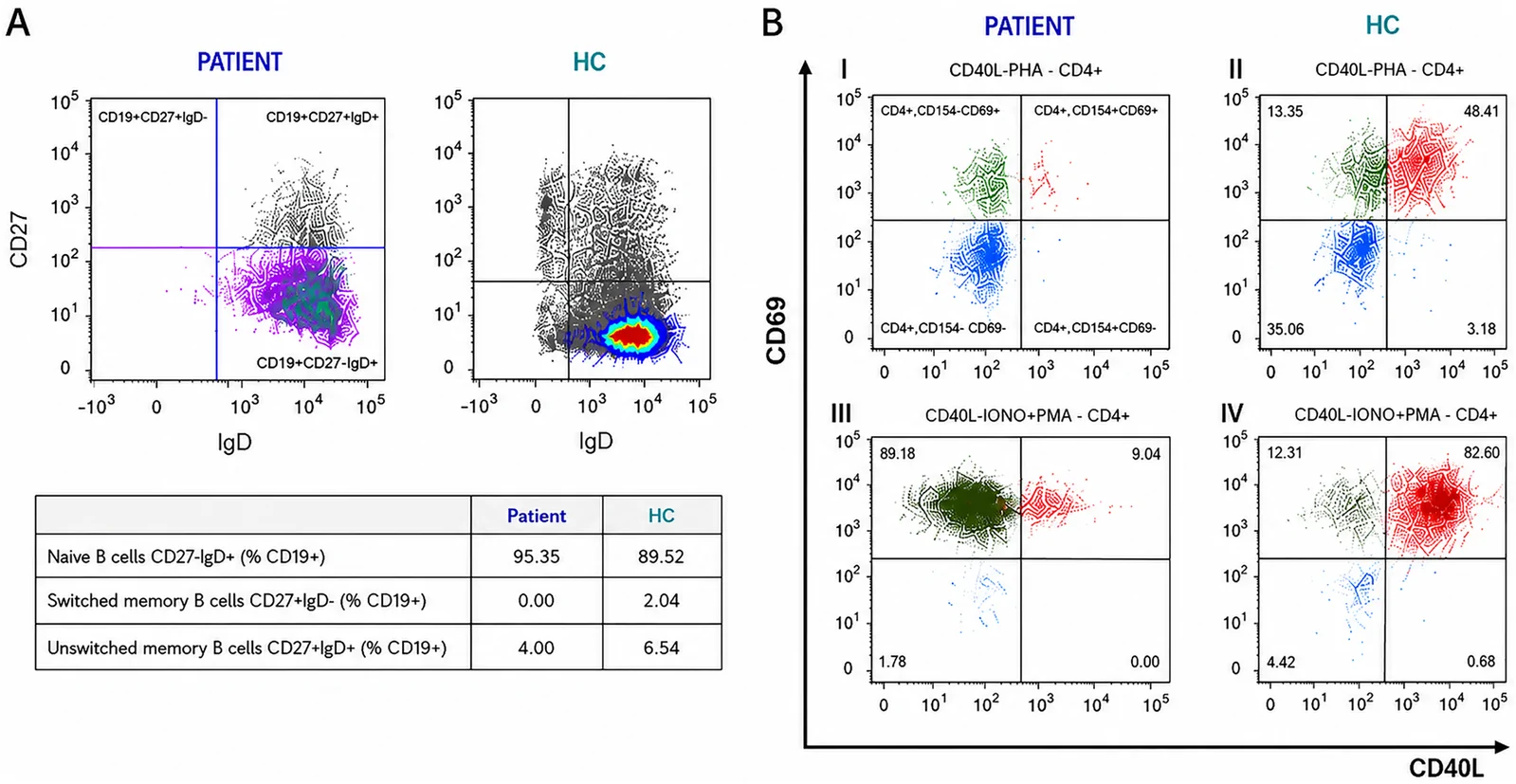
Flow cytometry analysis. **(A)** B-cell subpopulations of the patient and an age-matched healthy control (HC), with complete absence of switched memory B cells CD19+ CD27+ IgD− (upper left quadrants) in the CD40L-deficient patient. **(B)** Evaluation of CD40L expression on activated CD4+ T cells of the patient (I and III) and the healthy control (II and IV) after 24 h (I and II) and 4 h (III and IV) of T-cell stimulation with PHA and PMA-iono, respectively. CD69 was used as the activation marker. PHA, Phytohemagglutinin, used for prolonged T-cell stimulation to assess CD40L expression after 24 h; PMA-iono, Phorbol 12-myristate 13-acetate (PMA) and ionomycin, used for short-term T-cell stimulation to assess CD40L expression after 4 h.

Infectious causes were investigated using a multiplex polymerase chain reaction (PCR), and a culture was performed on the BAL fluid. Multiplex PCR was strongly positive for *P. jirovecii* (31,528,575 copies/mL), consistent with invasive infection. Given the association between opportunistic infections and secondary PAP, an immunologic evaluation was performed. The complete blood count showed a normal leukocyte differential and normal total lymphocyte count. Quantitative serum immunoglobulin measurements revealed low IgG levels (0.5 g/L; age-matched reference range 2.9–5.5 g/L) and low IgA levels (<0.1 g/L; reference range 0.1–0.4 g/L) with normal IgM levels (0.7 g/L; reference range 0.3–0.8 g/L) (15). Lymphocyte immunophenotyping, performed twice for confirmation, demonstrated a complete absence of class-switched memory B cells, leading to an evaluation of CD40L expression on activated T lymphocytes, which was markedly reduced and suggested CD40L deficiency (Figure 3). Complement studies were normal. Screening for secondary causes of immunodeficiency was negative. Finally, a next-generation sequencing-based primary immunodeficiency (PID) gene panel identified a hemizygous variant in *CD40LG* [c.137T>C, p.(Leu46Pro)], inherited from his mother, in whom the variant likely arose *de novo*. Considering the patient’s immunophenotype, the variant characteristics, and the segregation analysis, the variant was classified as likely pathogenic (see Discussion), suggesting X-linked immunodeficiency with hyper-IgM (XHIGM, #MIM 308230). Genetic causes of PAP were further investigated using a next-generation sequencing-based chILD panel (chILD_v5_042025). No pathogenic variants associated with congenital forms of PAP were identified. Other rare pediatric etiologies, including autoimmune and exposure-related hypersensitivity pneumonitis, were excluded; serum anti-GM-CSF antibodies were negative, and no relevant medication or environmental exposure was reported.

The clinical course was marked by progression to respiratory failure. Non-invasive ventilation was initially required, followed by invasive mechanical ventilation for 7 days because of severe hypoxemia and respiratory acidosis (initial oxygenation index 13.4). The *P. jirovecii* infection was treated with intravenous sulfamethoxazole-trimethoprim (20 mg/kg/day of trimethoprim) for 21 days. Hypogammaglobulinemia was substituted with intravenous human polyvalent immunoglobulins (IVIG). Gradual clinical improvement was observed, and oxygen therapy was discontinued after 1 month of hospitalization. A post-treatment reassessment, performed 2 weeks after completion of treatment and 6 weeks after the initial evaluation, showed improvement in pulmonary parenchymal abnormalities on chest CT but persistent basal interstitial and alveolar opacities (Figures 1C,D). Repeat BAL no longer supported active PAP, with unremarkable cytology and negative PAS staining, but remained positive for *P. jirovecii* on immunostaining, suggesting residual *Pneumocystis* infection. After the acute phase, sulfamethoxazole-trimethoprim was continued as prophylaxis (5 mg/kg/day of trimethoprim, 3 days per week). Regular IVIG replacement therapy was maintained. Additional preventive measures were implemented, including temporary exclusion from group childcare and reinforcement of hygiene and food safety measures at home. Given the underlying primary immunodeficiency of the patient, hematopoietic stem cell transplantation (HSCT) was considered the definitive therapeutic option, and the patient was registered on the transplant waiting list.

## 3 Discussion

Only a small number of pediatric patients with *CD40LG-*associated XHIGM syndrome and PAP have been reported. The majority of cases presented in infancy with severe respiratory disease (12). Previous reports have shown that PAP may occur with or without an identifiable infectious trigger, including *P. jirovecii* infection as a reversible cause in infants with immunodeficiency (13, 16, 17). More recently, Xu et al. reported on a 5-month-old boy with XHIGM in whom PAP was the initial manifestation without an identified infectious trigger; the patient recovered after whole-lung lavage (WLL), IVIG, cotrimoxazole prophylaxis, and allogeneic HSCT (12). In this limited body of literature, our 4-month-old patient with XHIGM, invasive *P. jirovecii* pneumonia, and PAP represents one of the youngest reported cases and further illustrates that PAP may be the initial manifestation of CD40L deficiency. These observations support the need to systematically investigate primary immunodeficiency, including defects of the CD40–CD40L pathway, when a PAP pattern occurs in early infancy, particularly in the presence of opportunistic infection. Pathophysiologically, PAP results from intra-alveolar accumulation of surfactant when the balance between its production by type II pneumocytes and its clearance by alveolar macrophages is disrupted (16, 17). GM-CSF-dependent differentiation and activation of alveolar macrophages are essential for surfactant catabolism, and impaired GM-CSF signaling is a well-established mechanism in several genetic and autoimmune forms of PAP (13). CD40 is expressed on antigen-presenting cells, including macrophages, and ligation by CD40L on activated CD4⁺ T cells enhances macrophage effector functions. CD40L deficiency may therefore impair macrophage activation through defective CD40 signaling, reducing surfactant clearance and predisposing patients to secondary PAP. Direct functional evidence in XHIGM remains limited, and further studies are required (1, 2, 5, 12, 13, 16). Opportunistic infections, particularly *P. jirovecii*, are frequent in both XHIGM and PAP and may further impair macrophage phagocytic activity and surfactant clearance, contributing to a multifactorial pathogenic process (9, 12, 14, 17). The clinical presentation in our patient suggests that intrinsic immune dysfunction and macrophage impairment are central to the disease, with *P. jirovecii* infection acting as an important cofactor rather than the sole cause of PAP. Susceptibility to *P. jirovecii* pneumonia varies according to the genetic form of hyper-IgM syndrome. It is primarily associated with CD40L and CD40 deficiencies, which are combined immunodeficiencies, unlike the other predominantly humoral forms. In CD40L deficiency (XHIGM), *P. jirovecii* infection may represent the first clinical manifestation and affects approximately 40% of patients. It may also occur in other primary immunodeficiencies affecting cell-mediated immunity, including severe combined immunodeficiency and idiopathic CD4T-cell lymphocytopenia (1, 3).

Immunologic evaluation in our patient showed low IgG and IgA levels with IgM at the upper limit of normal, an absence of class-switched memory B cells, and markedly reduced CD40L expression on activated T cells, a profile characteristic of CD40L deficiency. This finding is consistent with the role of CD40L–CD40 signaling in T cell-dependent B-cell activation, germinal center formation, and immunoglobulin class-switch recombination. Disruption of this pathway results in the hallmark pattern of reduced IgG, IgA, and IgE with normal or elevated IgM and decreased switched memory B cells in XHIGM (1, 2, 5, 12–14). Massive parallel sequencing has substantially improved etiologic diagnosis. In our patient, the chILD panel did not identify diagnostic variants associated with congenital surfactant metabolism disorders or GM-CSF receptor-related PAP. In contrast, the PID panel detected a hemizygous variant in *CD40LG* (located on chromosome Xq26.3), supporting a mechanism of secondary PAP (9, 12, 17).

*CD40LG* encodes the membrane glycoprotein CD40 ligand (CD40L/CD154/gp39) (4). The *CD40LG* variant detected in our patient [NM_000074.3: c.137T>C, p.(Leu46Pro)], located in exon 1, is a missense substitution that replaces a conserved leucine with a proline in the transmembrane domain of CD40L. The variant is absent from population databases (18) and, to the best of our knowledge, has not been described previously with XHIGM. The leucine-to-proline substitution is non-conservative, probably introducing structural constraint due to proline's cyclic pyrrolidine side chain determining high conformational rigidity. Consistently, the majority of *in silico* predictors confer a deleterious score, combining structural context and evolutionary conservation (19). Indeed, the variant is located at the very end of the TM domain, which forms an α-helix that could be destabilized by the introduction of a proline (Supplementary Figure 1) (20, 21). However, no *in vitro* functional data are available. Of interest in this context, there are no prolines in the TM domain (amino acids 23–46) and no proline substitutions in the healthy population in the TM domain (18), suggesting low tolerance for prolines at that site. Finally, a segregation analysis showed that the mother was heterozygous for the variant, but that it was absent in both maternal grandparents, suggesting that the variant arose *de novo* in the mother. Based on the combination of these phenotypic data, the variant characteristics, and segregation data, the variant was classified as likely pathogenic (ACMG class 4). The majority of pathogenic *CD40LG* variants have been reported in the extracellular domain, impairing CD40L interaction (22). Only a few variants located in the TM domain have been described. It has been suggested that TM variants are associated with a milder, atypical, and late-onset disease presentation given the pathophysiology of reduced surface expression of CD40L that, however, maintains some residual activity (22, 23). A literature review showed another case with a proline insertion in the TM domain with mild, late-onset disease (24). In contrast, our patient presented with early-onset disease and markedly reduced surface expression of CD40L on activated T cells. We hypothesize that this more severe phenotype may be related to the location of the variant at the very end of the TM domain.

This case illustrates the importance of integrating clinical, immunologic, and genetic data when interpreting variants in PID genes, allowing translation of a descriptive genetic finding into a clinically actionable diagnosis, which informs prognosis and therapeutic decisions, including HSCT. In children, treatment strategies for PAP remain largely empirical because of limited evidence and are primarily based on expert opinion. Management depends on the underlying etiology and the severity of respiratory impairment, with the goal of improving gas exchange and controlling the underlying condition (9, 10). WLL is generally considered the first-line therapy for autoimmune or idiopathic PAP with significant hypoxemia. Although it can rapidly improve oxygenation, repeated procedures are often required, and efficacy is variable, particularly in congenital surfactant disorders. The procedure is technically demanding and carries potential risks for very young infants, even at experienced centers (25–27). Pharmacologic approaches are increasingly used as alternatives or adjuncts in selected cases. Inhaled GM-CSF has shown benefit in autoimmune PAP by reducing the need for repeated WLL, whereas rituximab and plasmapheresis are primarily reserved for refractory autoimmune disease (28–30). Their efficacy in other forms of secondary PAP, particularly in children, remains uncertain, and none can currently be considered a standard therapy. Lung transplantation is reserved for rare end-stage hereditary cases with irreversible respiratory failure. In secondary PAP, treatment primarily focuses on control of the underlying disorder (9, 10). Our patient’s respiratory status improved with ventilatory support and targeted treatment of the underlying infection, prompting us not to perform WLL. High-dose trimethoprim-sulfamethoxazole for invasive *P. jirovecii* pneumonia, combined with IVIG replacement therapy, led to progressive clinical recovery and avoided a high-risk invasive procedure in a 4-month-old infant. Long-term treatment must address the underlying CD40L deficiency. Standard care for XHIGM includes regular immunoglobulin replacement therapy, *P. jirovecii* prophylaxis, and strict infection prevention measures (1, 2). Immunoglobulin therapy reduces bacterial infections but does not fully prevent opportunistic infections or long-term complications. Despite optimized supportive care, patients remain at risk of chronic lung and liver disease, autoimmune manifestations, hematologic abnormalities, and malignancy (1, 3). Allogeneic HSCT is therefore considered the only curative therapy, as it restores CD40L–CD40 signaling, normalizes immunoglobulin production, and reduces the risk of severe infections and long-term morbidity, particularly when performed before irreversible organ damage occurs (1, 5). In a recent systematic review, HSCT was performed in approximately 45% of patients with CD40–CD40L pathway defects and achieved cure in 73% of transplanted patients. The overall mortality was 21%, increasing to 25% among non-transplanted patients, suggesting a survival advantage with early HSCT (5). Moreover, Mitsui-Sekinaka et al. reported improved outcomes when HSCT was performed before 5 years of age, likely due to limiting cumulative infections and organ damage (31, 32).

Several limitations should be acknowledged. As this is a single case, causal inference remains limited, and the respective contributions of XHIGM, pneumocystosis, and PAP to the observed lung pathology cannot be fully disentangled. Long-term follow-up after HSCT and immune reconstitution will be necessary to determine whether PAP resolves definitively or recurs. Although *in silico* predictions, segregation analysis, and immunologic findings support the pathogenicity of the variant, no variant-specific functional *in vitro* data are currently available. Additional studies are needed to confirm its precise molecular effect. Lung histology could have provided additional mechanistic insight but was not performed because bronchoalveolar lavage with PAS staining and microbiological analysis provided a rapid, less-invasive diagnostic approach, consistent with the family's preference to avoid a surgical lung biopsy.

## 4 Conclusions

This case highlights pulmonary alveolar proteinosis as a rare early manifestation of CD40L deficiency and illustrates how invasive *P. jirovecii* infection may contribute to PAP pathogenesis in infants with XHIGM syndrome. In young children presenting with PAP, particularly when associated with opportunistic infection, systematic evaluation for underlying primary immunodeficiency is essential. Comprehensive assessment integrating clinical findings, immunologic profiling, and genetic analysis is critical for interpreting novel *CD40LG* variants and establishing a definitive diagnosis. We reported a previously undescribed *CD40LG* variant, c.137T>C, p.(Leu46Pro), classified as likely pathogenic, thereby expanding the mutational spectrum of XHIGM syndrome. Early recognition of this condition allowed prompt initiation of appropriate antimicrobial and immunoglobulin therapy and supported the timely consideration of hematopoietic stem cell transplantation as the only curative treatment.

## Data Availability

The datasets presented in this study can be found in online repositories. The names of the repository/repositories and accession number(s) can be found in the article/Supplementary Material.
