# Supplementary material for: Pulmonary alveolar proteinosis and Pneumocystis jirovecii infection in an infant with hyper-IgM syndrome caused by a novel CD40LG variant: a case report
Source: Front Pediatr. 2026 Aug 11;14:1913335. doi: 10.3389/fped.2026.1913335 (PMC13504799; doi:10.3389/fped.2026.1913335)
Supplement: Supplementary file 1 [file Supplementaryfile1.docx]

Supplementary Material

# 1 Supplementary Data

/

# 2 Supplementary Figures and Tables

**Supplementary Figure 1. Structural and phylogenetic analysis of the CD40LG p.(Leu46Pro) variant.**

(A) Predicted 3D structure of wild-type CD40L obtained using AlphaMissense. Leu46 is located within the transmembrane α-helix (yellow), where no proline residue (green) is present in the wild-type transmembrane helical domain. This localization supports the structural relevance of the p.(Leu46Pro) substitution. (20)
(B) Phylogenetic conservation of Leu46 across species. Display generated using Clustal Omega Multiple Sequence Alignment. (21)

A	B
